# Human Chorionic Gonadotrophin: New Pleiotropic Functions for an “Old” Hormone During Pregnancy

**DOI:** 10.3389/fimmu.2020.00343

**Published:** 2020-03-13

**Authors:** Virginie Gridelet, Sophie Perrier d'Hauterive, Barbara Polese, Jean-Michel Foidart, Michelle Nisolle, Vincent Geenen

**Affiliations:** ^1^GIGA-I3 Center of Immunoendocrinology GIGA Research Institute, University of Liege, Liege, Belgium; ^2^Center for Assisted Medical Procreation, University of Liège, CHR Citadelle, Liège, Belgium; ^3^Laboratory of Tumor and Development Biology, University of Liège, Liège, Belgium; ^4^Department of Obstetrics and Gynecology, CHR Citadelle, University of Liège, Liège, Belgium

**Keywords:** hCG, implantation, pregnancy, immunology, miscarriages, autoimmunity

## Abstract

Human chorionic gonadotrophin (hCG) is the first specific molecule synthesized by the embryo. hCG RNA is transcribed as early as the eight-cell stage, and the blastocyst produces the protein before its implantation. hCG in the uterine microenvironment binds with its cognate receptor, luteinizing hormone/choriogonadotropin receptor (LHCGR), on the endometrial surface. This binding stimulates leukemia inhibitory factor (LIF) production and inhibits interleukin-6 (IL-6) production by epithelial cells of the endometrium. These effects ensure essential help in the preparation of the endometrium for initial embryo implantation. hCG also effects angiogenic and immunomodulatory actions as reported in many articles by our laboratories and other ones. By stimulating angiogenesis and vasculogenesis, hCG provides the placenta with an adequate maternal blood supply and optimal embryo nutrition during the invasion of the uterine endometrium. The immunomodulatory properties of hCG are numerous and important for programming maternal immune tolerance toward the embryo. The reported effects of hCG on uterine NK, Treg, and B cells, three major cell populations for the maintenance of pregnancy, demonstrate the role of this embryonic signal as a crucial immune regulator in the course of pregnancy. Human embryo rejection for hCG-related immunological reasons has been studied in different ways, and a sufficient dose of hCG seems to be necessary to maintain maternal tolerance. Different teams have studied the addition of hCG in patients suffering from recurrent miscarriages or implantation failures. hCG could also have a beneficial or a negative impact on autoimmune diseases during pregnancy. In this review, we will discuss the immunological impacts of hCG during pregnancy and if this hormone might be used therapeutically.

## Introduction

In its first days of development, the trophoblast secretes an important hormone: the chorionic gonadotrophic hormone (hCG). hCG is going to have a series of actions in the survival of the embryo, the best known of which is progesterone secretion maintenance by the corpus luteum (1).

hCG is a glycoprotein hormone of 36–40 kDa. It is composed of two subunits, α and β, linked with a noncovalent bond. The α subunit, composed of 92 amino acids, is encoded in chromosome 6 and is common in various hormones of the glycoprotein family including luteinizing hormone (LH), thyroid-stimulating hormone (TSH), and thyroid-stimulating hormone (TSH). The β subunit, which is different for each hormone, is encoded on different genes on chromosome 19 (LH, hCG, and TSH) or on chromosome 11 (FSH). The β subunit of hCG is encoded in six different but very similar genes located in a group of genes on chromosome 19 (2). The β subunit of hCG is, with 145, the β subunit with the highest number of amino acids but also the largest glycosylated domain. This gives the hCG greater stability and facilitates its rapid secretion. Unlike other glycoprotein hormones that are synthesized by the anterior lobe of the pituitary gland, hCG is not only produced by the trophoblast (and mainly by the syncytiotrophoblast) but also by malignant tumors. hCG contains four N-linked oligosaccharides and four O-linked oligosaccharides.

hCG's structure is similar to that of LH, but unlike LH, hCG exists in several forms, known as classical hCG, hyperglycosylated hCG, and the free β unit of hyperglycosylated hCG (3). Each of these four molecules has different physiological functions. Chorionic gonadotropins only exist in primates (in humans, this is hCG) and in equines (named eCG for equine CG). In mice, LH could play the same role as hCG and is secreted early by the embryo (4).

## hCG Isoforms

In addition to conventional hCG, there is a highly glycosylated hCG variant, which is hyperglycosylated hCG (hCG-H). Its β subunit has four oligosaccharide-linked Os instead of two in classical hCG (5). This variant is massively produced during the first trimester of pregnancy by the extravillous cytotrophoblasts, the form of hCG that is the most massively present during the very beginning of pregnancy. It represents 87% of the total hCG in the third week of gestation and 51% during the fourth week. Then, it decreases rapidly until it completely disappears from the maternal blood circulation at the end of the first trimester (6). hCG-H is known to have an autocrine action rather than an endocrine action, decreasing the apoptosis of trophoblast cells (7) and inducing the implantation of the embryo (8) and trophoblastic invasion (9). It is also massively secreted by choriocarcinomas and germ cell tumors (5, 9, 10). A team suggested recently that hCG-H is functionally similar to hCG, although it has lower potency for luteinizing hormone/choriogonadotropin receptor (LHCGR) activation (11). This result is controversial with other results but not impossible (12, 13). hCG-H might act through different receptors.

hCG-H monitoring is useful for predicting Down's syndrome (9), preeclampsia (14), therapeutic response to trophoblastic diseases, and pregnancy predictions performed in *in vitro* fertilization (15).

The free β subunit of hCG would also act like an antagonist through the transforming growth factor beta (TGF-β) receptor (16, 17) and is enabled to activate LHCGR **(**11). Like hCG-H, this subunit would have a promotive action on cancer.

The sulfated hCG produced by the pituitary gland is hardly detectable during the menstrual cycle. It is secreted in parallel with LH during the cycle and is concentrated at approximately one-fifth of the LH concentration (18–20). While these levels are low, sulfated hCG is exactly 50 times more potent than LH (21). Thus, sulfated hCG could perform comparable work with LH in stimulating androstenedione production during the follicular phase of the cycle as well as stimulating ovulation and corpus luteum formation. During the luteal phase, it may help stimulate progesterone production (18–21).

## hCG Secretion

hCG is one of the first molecules secreted by the embryo. Its RNA is transcribed as early as the eight-cell stage (22), and the blastocyst produces the protein before implantation (23, 24). The syncytiotrophoblast highly produces this hormone after implantation (25). Significant concentrations of hCG can already be measured in the maternal blood 10 days after ovulation. hCG concentration reaches its peak during the first trimester of pregnancy. It occurs around the 10th of gestation and can be measured 75,000 IU/L. Afterwards, the level decreases gradually to the 19th week. Its remains basal until the end of the pregnancy, ~15,000 IU/L. This rate remains higher than in nonpregnant women (26, 27). It has been recently shown that during *in vitro* fertilization (IVF) treatments, faster-growing blastocysts produced significantly higher serum β-hCG concentrations 9 days after transfer than slower-growing blastocysts in fresh cycles, but the difference was not significant by day 16 after transfer (28).

Macrophages can regulate excess hCG, known to have teratogenic effects on fetal tissues. Human fetal tissue macrophages are proposed to incorporate and destroy hCG in a time-dependent manner, which protects fetal gonadogenesis from the deleterious effects of hCG (29, 30). Specifically, Katabuchi and his team have recently shown that hCG induces the formation of vacuoles in human monocytes. With these vacuoles, they look like fetal Hofbauer cells. They hypothesize that Hofbauer cells, and more particularly their vacuoles, would be involved in the protection of fetal tissues against unusually high concentrations of hCG (31).

Abnormalities in the production and the circulating levels of the several glycoforms of hCG throughout specific periods of gestation and in the relative variations have been associated with a large array of pregnancy complications, such as miscarriages (32), fetal chromosomal anomalies (33), preeclampsia (34, 35), disturbances in fetal growth and development (36), and gestational trophoblastic diseases (37). The serum β-hCG level predicts biochemical/clinical pregnancy and singleton/multiple pregnancy with robust sensitivity and specificity (38).

Emerging evidence suggests that prenatal exposure to selected endocrine disrupting chemicals (EDCs) have a deleterious impact on the fetus and long-lasting consequences in adult life as well. Several reports have shown that *in vitro* effects of commonly found EDCs, particularly bisphenol A and para-nonylphenol, can alter hCG production, and through this action, it might exert their fetal damage [reviewed by Paulesu et al. (39)].

hCG (or its alpha subunit or beta subunit) is also secreted by gestational trophoblastic neoplasia. It includes malignant invasive mole, choriocarcinoma, and rare placental site trophoblastic and epithelioid tumors (40). hCG can be found in testicular cancer. Gestational choriocarcinoma and testicular cancer have been routinely curable for over 50 years and have cure rates approaching 95 and 85%, respectively. In contrast, hCG production by cancers, aside from these two types, is generally associated with a worsening of the prognosis, like in lung, liver, or ovary cancers (41, 42). Immunization to hCG in experimental models has been shown to have antitumor effects (43–45).

## hCG Actions

**Figure 1:**
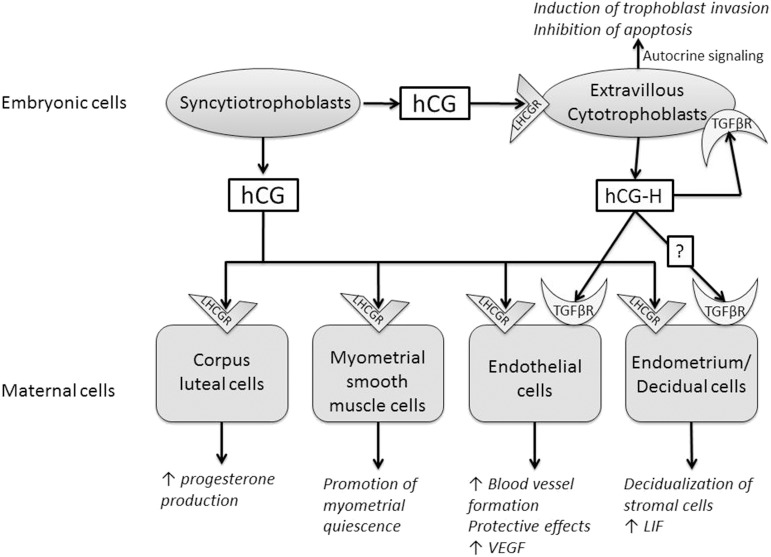
Summary of the paracrine and endocrine actions of human chorionic gonadotrophin (hCG) and hyperglycosylated hCG (hCG-H). These two molecules act through two different receptors, luteinizing hormone/choriogonadotropin receptor (LHCGR) and transforming growth factor beta receptor (TGFβR).

In 1920, Hirose showed a hormonal link between a hormone produced by the placenta and the production of progesterone by corpus luteum's cells (3, 46). This hormone has been called gonadotropic chorionic hormone. Stimulation of progesterone production by the corpus luteum has long been the only known function of hCG (Figure 1). This hormone induces also the upregulation of aromatase expression and estradiol production in human granulosa lutein cells. This effect might be mediate by amphiregulin (47). hCG strongly stimulates the expression of its own receptor in human luteal cells (48).

Three independent teams have shown that the preimplantation blastocyst secretes hCG into the uterine space that will bind to its LHCGR receptor on the deciduous surface. In response, the decidua prepare for implantation (49–51). The stromal cells undergo the decidualization under the effect of hCG, after which they secrete prolactin (52). hCG also increases the secretion of leukemia inhibitory factor (LIF) and decreases the secretion of interleukin-6 (IL-6) by endometrial cells, molecules known for their influence on embryo implantation (50). It promotes the differentiation of cytotrophoblasts into syncytiotrophoblasts (53), and it can regulate prostaglandin synthesis (54) and the formation of cyclic AMP (cAMP) (55). A study has shown that different forms of hCG might stimulate trophoblastic invasion independently of the classical hCG receptor, LHCGR (56). The glycosylation of hormones has a direct influence on their bioactivity. Hyperglycosylated hCG would be more beneficial than conventional hCG for the implantation (8). Further studies are required to clarify whether this hCG-H effect on endometrial stromal cells involves LHCGR or TGFβ receptor (TGFβR) or both (57).

hCG also has angiogenic actions as discussed in two papers from our laboratory (58, 59). hCG increases blood vessel formation and the migration and maturation of pericytes in different *in vitro* and *in vivo* models (60–63). It also has a positive impact on the secretion of vascular endothelial growth factor (VEGF), a well-known molecule of angiogenesis (60, 64). Recently, a study has shown that hCG regulates VEGF through the activation of nuclear factor kappa B (NF-κB) in luteal angiogenesis (65). hCG has protective effects on vascular endothelial cells against oxidative stress through inhibition of apoptosis, activation of cell survival signaling, and mitochondrial function retention (66).

hCG-H also displays a potent angiogenic effect. However, hCG-H induces angiogenesis regardless of LHCGR signaling pathways (13, 67). The antiapoptotic action of hCG-H would also be achieved independently of LHCGR. The specific receptor(s) activated by hCG-H on trophoblasts cells and, potentially, on various decidual cells has/have not been fully identified (57). Berndt et al. demonstrated that hCG-H displayed a potent angiogenic effect by interacting with TGFβR (more precisely TGFβRII). They eliminated the angiogenic effect of hCG-H by the addition of SB431542n, the antibody against TGFβRII, in the culture medium of their model. It was confirmed in LHCGR-knockout mice (13). Several groups implicated hCG-H in promoting growth and invasion of placental and germ cell malignancies through the TGF-β signaling pathway by utilizing potential autocrine interactions (17, 68). Structural similarity between hCG-H and TGFβ supported this idea of interaction between hCG-H and TGFβRII. They share a unique four-peptide cysteine knot structure identified in several cytokines that collectively form the cysteine knot growth factor family (67, 69, 70). Interestingly, a study reported a woman with an inactive mutant LHCGR who maintained a normal pregnancy after becoming pregnant with ovum donation. This suggest that the maintenance of pregnancy through LHCGR activation is not unavoidable and that, during pregnancy, the main hCG effect may be mediated by other mechanisms than LHCGR activation (71).

The team of Gallardo has suggested that the striking overlapping of hCG and Heme oxygenase-1 (HO-1) functions in pregnancy could indicate that hCG hormonal effects are mediated by HO-1 activity, which may be affected by a HMOX1 polymorphism in humans (72). HO-1 regulates angiogenesis and vasculogenesis, and trophoblasts proliferation, migration, and invasion, thus contributing to the adaptive changes in the uterine circulation to pregnancy.

hCG and hCG-H are therefore considered proangiogene molecules. By stimulating angiogenesis and vasculogenesis, they allow the placenta to have adequate maternal blood supply during functional endometrial invasion and optimal fetal nutrition.

A study investigated the effects of different doses of hCG on hCG receptor-immunoreactive neuron density in the prefrontal cortex and cerebellum of a rat model of stretozotocin-induced Alzheimer's disease (73). hCG administration resulted in a significant dose-dependent increase in the number of hCG receptor-ir neurons in the prefrontal cortex and cerebellum (74). The same group showed that hCG attenuates amyloid-β plaques induced by streptozotocin in the rat brain by affecting cytochrome c-ir neuron density (75). They conclude that hCG might be useful in patient with Alzheimer's disease to prevent the congophilic Aβ plaque formation and decrease cytochrome c-immunoreactive neuron density in the brain.

## Immunological Actions of hCG

The immunomodulatory properties of hCG are numerous and important for maternal tolerance of the embryo (59). The activation of the maternal immune system tolerance appears essential for the embryonic development and the implantation (76).

CD4+ T cells can be classified into the following subsets: T helper (Th) 1, Th2, Th17, and regulatory cells T (Treg) according to their functions. One study indicated that immunity in patients suffering with recurrent miscarriages is dominated by the Th1/Th2 hypothesis (77). However, the Th1/Th2 paradigm alone is not enough to explain the mechanism by which the fetus is rejected by maternal immune cells. The Th1/Th2 paradigm has been extended to the Th1/Th2/Th17 and Treg cell paradigm. Th17 cells and Treg have been described as lymphocyte subsets that are different from Th1 and Th2 cells. These are known to play a major role in the development of autoimmune diseases and infection. Previous studies have shown that Th17/Treg imbalance can be associated with recurrent spontaneous abortion (78, 79). While most studies show that hCG has a suppressive effect on T-cell proliferation (80), trophic effects of hCG have also been reported (81). Evidence exists of a potential intersection between the hCG and the T cell receptor (TCR) signal. In a contradictory manner, hCG encourages trophoblast invasion and interstitial theca cell proliferation by overmodulating extracellular-regulated kinase (ERK) and AKT signals (82, 83); leptin production by hCG requires a dialogue between cAMP and p38 signaling pathways in the syncytiotrophoblast (84). Gestational trophoblastic neoplasias strongly express programmed cell death ligand 1 (PD-L1), a protein expressed by T cells activated. The team from Ghorani describes the curative treatment of women with chemotherapy refractory choriocarcinoma with Pembrolizumab (PD1 immunotherapy) (40). This paper shows the impact that removal of T cell regulation has on the interface between T cell and trophoblast cells and further strengthens the debate regarding the immunosupressive pregnancy environment in part resulting from hCG.

Furthermore, hCG has different effects on CD4+ T cells. During the 1970s, it was suggested that hCG might have an effect on maternal lymphocytes (85). Since then, it has been shown that hCG has a positive impact on the proliferation of CD4+25+ T cells and that it attracts these cells to the endometrium in early pregnancy (86, 87). Immune cells located at the implantation site actively contribute to embryo implantation (88, 89). hCG increases the frequency of murine Treg cells *in vivo* and decreases their suppressive activity *in vitro* (90).

hCG increases the presence of regulatory T cells and increases the level of IL-1beta in mice (91). This hormone appears to play a key role as a tolerance modulator during pregnancy (90). A recent study shows that hCG inhibits the expression of CD25 and CD28 on the surface of naive T cells (CD45RA+) and the expression of CD25 on memory T cells (CD45R0+). Apparently, hCG promotes the differentiation of memory T cells by increasing the expression of CD45R0+ but reduces their functional activity toward fetal antigens through a competitive process. hCG also increases the production of IL-2 by naive and memory T cells. This hormone is therefore involved in the regulation of these T cells (92).

By modulating the balance between inflammatory-type Th1 cells and anti-inflammatory-type Th2, the hCG plays a fundamental role in the implantation of the embryo (59, 93).

hCG has a positive impact on uterine natural killer (uNK) cells, important leukocyte cells in the nongestating uterus that act on the establishment and maintenance of embryo implantation in women and mice (94–96). hCG regulates the proliferation of uNKs (97) in a dose-dependent manner *in vitro* (98). These cells do not express LHCGR, and hCG would act directly on these cells through another receptor, mannose receptor (98), which is expressed by the uNK. uNKs participate in the remodeling of spiral arteries, a crucial vascular modification for the vascularization of the placenta, which guarantees a sufficient supply to the fetus (99). They also secrete proangiogenic factors such as members of the VEGF family (100).

In a murine model, different teams demonstrated an inhibitory effect of hCG on bone-marrow-derived dendritic cells (DCs) as well as on peripheral and local (decidual) DCs, therefore supporting the idea that hCG supports a tolerogenic rather than an immunogenic DC phenotype. Furthermore, hCG influence the differentiation and function of DCs, decreasing their ability to stimulate T-cell proliferation (93, 101, 102).

hCG acts on other immune cells, like monocytes, by promoting their function and secretion of IL-8 (103) and also induces the functions of macrophages (104). By stimulating the function of macrophages, hCG cleans the endometrium by purifying apoptotic cells and fighting possible infections, which are two important mechanisms in the maintenance of pregnancy.

It has been shown that hCG could increase the ability of trophoblast cells to invade the extracellular matrix *in vitro*, which is accompanied by an increase in the expression of matrix metalloproteinase (MMP)-2, MMP-9, and VEGF and a decrease in the expression of tissue inhibitor matrix metalloproteinase (TIMP)-1 and TIMP-2. Peripheral blood mononuclear cells (PBMCs) support *in vitro* embryo invasion, and hCG enhances the effects of PBMCs (105).

An *in vitro* study supports the hypothesis that hCG is not a regulator of cell damage from peripheral blood dendritic cells (PBDCs). Nevertheless, in an inflammatory context, hCG seems to maintain the delicate balance between plasmoid dendritic cells and myeloid dendritic cells (MDCs) and seems to retain a tolerogenic MDC1profile, which might contribute to maintaining tolerance (106).

The administration of hCG could have an impact on the cytokine profile expressed by the endometrium (107). hCG directly or indirectly influences the genetic expression of several cytokines in cell signaling, proliferation, apoptosis, immunological modulation, tissue remodeling, and angiogenesis in endometrial stromal cells (108). A study was performed in a 3D cell culture model to demonstrate that hCG administration significantly alters the secretion of several cytokines in epithelial cells, stromal cells, and both cell types together (109). hCG inhibits the expression of tumor necrosis factor alpha (TNFα) and interferon gamma (IFN-γ) in the maternal/fetal interface and decreases the rate of resorption in abortive mouse models (110). Bai et al. cultured *in vitro* PBMCs with different concentrations of hCG and showed that hCG significantly inhibited IL-6 and TNFα messenger RNA (mRNA) expression, indicating that hCG could inhibit the production of proinflammatory cytokines (111).

Control of complement system's activation in the feto-maternal environment seems critical for embryo development. One study has shown that hCG plays a role in this complement control, particularly on decay accelerating factor (DAF) and C3 protein, in *in vitro* and *in vivo* models (112).

Therefore, the hCG has an important immunomodulatory function, and its effects on the cells uNK and Treg (two major cell populations in the maintenance of pregnancy) demonstrate the crucial role of this embryonic signal as an immune regulator in the course of pregnancy. For more information on the regulation of these immune cells by hCG, you can read the article of Schumacher and Zenclussen (113).

## hCG and Autoimmune Diseases

Pregnancy, which encourages a Th2-like environment, should encourage the production of antibodies making this type of disease more aggressive while improving Th1/Th17-related diseases.

Indeed, pregnancy is associated with an improvement in autoimmune disease symptoms associated with a Th1 profile (114, 115). The administration of hCG prevents (or decreases the severity) T-cell mediated autoimmune diseases in mice and humans (86, 116–118).

Unfortunately, the effect of pregnancy is rather deleterious for diseases with a Th2 cytokine profile. Pregnancy is believed to constitute a Th2 environment, where heightened hormonal levels may influence disease and promote its effects (119, 120). Systemic lupus erythematosus (SLE) is thought to be a disease induced by autoantibodies. Pregnancy-associated flares have been reported in certain studies (114). Estrogens and prolactin have deleterious effects on SLE (121, 122), where progesterone and testosterone have beneficial effects (122, 123). SLE is characterized by broad-spectrum antibody responses against autoantigens (124, 125). Higher levels of hCG have been reported in pregnant women with lupus (126), and nonpregnant patients with lupus also have high levels of hCG in their blood (127). A clinical case has been reported to show the appearance of SLE in three patients receiving hCG to induce ovulation (128). These results suggest that selective transductive, proliferative, and differentiative effects of hCG on adaptive immune cells may drive autoreactive responses in lupus environment and may also potentially provide insights into the association between the presence of higher hCG levels (or the administration of hCG) with the presence (or appearance) of humoral autoimmunity (129).

Regulatory B cells can modulate the immune response by creating an immunotolerant environment in autoimmune diseases and infections (130, 131). Several studies have suggested that B10 cells and Breg IL-35+ cells may play a role in autoimmune diseases and during pregnancy (132, 133). The combination of hCG and IL-35 induces the amplification of Breg IL-35+ and B10 cells that play a vital role in peripheral regulation during pregnancy (134). This phenomenon could have an influence on autoimmunity during pregnancy. hCG has also been identified as an important factor regulating phenotypes and the production of B-cell antibodies (135, 136). B1a B cells produce autoantibodies and proliferate in response to hCG (137).

Injections of hCG in patients are associated with ovarian hyperstimulation syndromes (OHSSs). (138, 139), a condition that is characterized by thrombosis that itself is related to the presence of antiphospholipid antibodies. In addition, increased levels of hCG have been associated with preeclampsia (74, 140), another thrombotic disease with possible autoimmune origins (141, 142). A study in a pregnant mouse model with lupus shows that hCG increases autoimmune responses *in vitro* and *in vivo*, events that are correlated with high responses at the cytokine level. In this study, hCG had a stimulating effect on the expression of CD40 and CD86 on B cells (129). On the contrary, after repeated hCG injections in nonobese diabetic mice, an induction of indoleamine 2,3-dioxygenase in dendritic cells could be observed. It resulted in an inhibition of autoreactive T cells and the prevention of diabetes onset (143). hCG can have effects that accumulate or decrease diseases of the immune system. These effects could become visible in a Th2 environment like pregnancy.

## Autoimmunity to hCG?

Cases of anti-hCG autoimmunity have been described, where patients have a history of repeated attempts to perform unsuccessful IVF treatments (144). The beta subunit of hCG is not immunogenic in women (145). Currently, the hCG vaccine is the only vaccine against pregnancy that has passed Phase II clinical trials. The Talwar et al. study provides evidence that persistent anti-hCG antibodies prevent pregnancy; in 1,224 sexually active women, only 1 pregnancy was observed. The authors also reported that fertility was restored when anti-hCG levels in the serum dropped below 35 ng/ml and thus that the effects of the vaccine were reversible (146). Contraception is achieved without impacting ovulation or menstrual disturbance.

Anti-hCG antibodies have been observed in young men who have been treated with exogenous hCG. The possible role of these particular antibodies in establishing and maintaining infertility is unclear. Hearn et al. reported that marmoset embryos exposed to anti-hCG immunoglobulins did not implant (147). Immunization against hCG has been shown to block fertility in baboons and rhesus monkeys (148). Anti-hCG vaccination studies in women provide evidence that a high level of antibodies may be a cause of fertility failure (149). Nevertheless, it is clear that not all women will develop autoimmunity to hCG after pregnancy or assisted reproductive treatment. In this context, a structural alteration, due to a mutation in one of the beta-hCG genes and/or a functional abnormality of the immune system, could be implicated. In one case of infertility described with hCG immunization, pregnancy was achieved as a result of plasmapheresis treatment to detoxify the patient's body (144). Immunotherapy could be beneficial for patients who suffered from repeated implantation failures (RIFs) previously. The problem would be the assessment of autoimmunity to hCG.

## Infusion of hCG During Embryo Transfer

RIF is a source of great frustration for patients. They concern patients who have carried out several IVF treatments with embryo transfers that have failed in pregnancy. The RIFs have a variable definition, but the most generally used definitions correspond to transfers of three or more embryos or 10 or more embryos of good quality in a patient and no pregnancy was obtain. The causes of RIF include many factors of maternal or fetal origin (150). Unfortunately, in many patients, the origins of RIF are not identified. A team administered intracerebral PBMCs previously activated by hCG, and the percentage of pregnancy was better when these activated PBMC cells were administered in patients with RIF (151, 152).

Intrauterine hCG infusion has been proposed to improve the success rates of embryo transfer during IVF treatments. hCG plays an important role in synchronizing fetal and endometrial developments. Many studies in recent years have investigated the impact of this intrauterine hCG administration before embryo transfer in patients with repeated implantation failures. The results of these studies are controversial (153–155). The differences in design and population can explain the contradictory results. Endometrial receptivity may also affect the effectiveness of hCG administration. Further evidence from multicenter, randomized controlled trials are needed to confirm the potential therapeutical intervention of hCG. Several meta-analyses seem to show a positive effect in RIF patients (156, 157).

Since embryos can be transferred at the end of the cleavage stage or at the blastocyst stage, the clinical effect of hCG administration in the uterus may be different even if performed just before the embryo transfer. The influence of this administration on the pregnancy rate was controversial when it took place before the transfer of cleaved embryos (158–162), while there was no improvement in the rate when administration was performed before embryo transfer at the blastocyst stage (154, 163).

**Figure 2:**
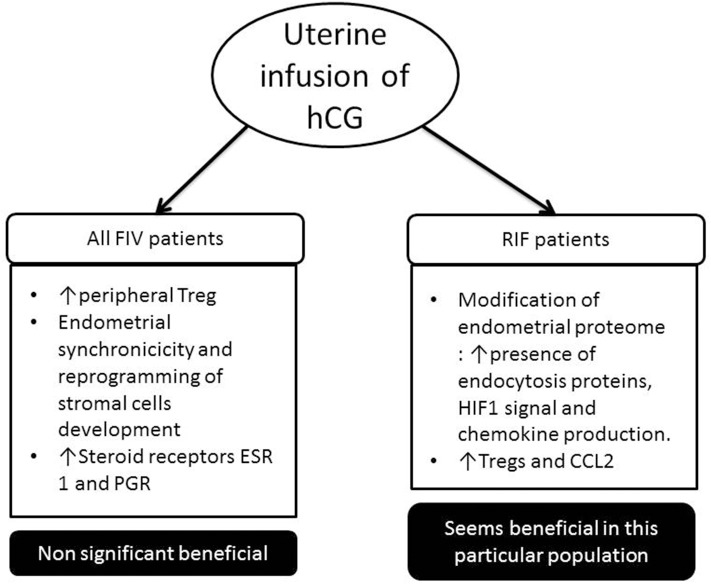
Impact of uterine infusion of human chorionic gonadotrophin (hCG) in classical *in vitro* fertilization (IVF) patients and in patient suffering from repeated implantation failure (RIF) (at least three embryos have been transferred in these patients, and no pregnancy was observed). In classical IVF patients, in most studies, there has been no significant increase in pregnancy rate following the introduction of hCG intrauterine. Whereas in the RIF population, several studies show that it is beneficial for the implantation of the embryo.

Following these clinical studies, various authors have studied the impact that this administration of hCG could have on the physiological level (Figure 2). One team showed that the percentage of peripheral Treg was increased compared to control patients when intrauterine hCG was administered (164). hCG was administered in the uterus from oocyte donors 3 days after their puncture to observe changes in the endometrium before embryo implantation. Infusion of hCG has been associated with endometrial synchronicity and reprogramming of stromal cell development following ovarian stimulation. Steroid receptors, estrogen receptor I (ESR I) and progesterone receptor (PGR), were significantly higher in treated vs. control patients (165).

Infusion of hCG during the implant window in a nonhuman primate model increased the expression of glycodelin by endometrial cells (166). Glycodelin is a protein secreted by the glandular portion of the endometrium that is expressed during and after the implant window (167). This protein has been suggested to play a role as an immunomodulator for the prevention of fetal allograft rejection by maternal cells (168, 169).

One study showed for the first time that the endometrial proteome composition of RIF patients differs from fertile controls during the window of implantation. The *in vivo* infusion of hCG into the uterine cavity of RIF patients stimulated the presence of endocytosis proteins, hypoxia-inducible factor-1 (HIF1) signal, and chemokine production (170). They hypothesize that the intrauterine infusion of hCG before an embryo transfer could improve the chemokine triggered embryo-endometrial dialogue and intensify the angiogenesis and immune response. Another team showed that infusion of hCG increased the endometrial Tregs and CCL2 expression in RIF patients, while the Tregs migration was blocked with CCL2 small interfering RNA (siRNA) or CCR2 antagonist treatment *in vitro* (171). We believe that intrauterine infusion of hCG might be a new therapy for Treg-decreased RIF patients, which need to be explored in a larger prospective study.

## hCG and Miscarriages

hCG and progesterone were analyzed concomitantly to determine whether their levels would be able to rapidly predict whether or not doubtful early pregnancy will continue, and the results provided a 48-h diagnosis of viability in 41.1% of patients (172). Another study shows that hCG and progesterone levels 14 days after oocyte retrieval may be predictive for continued pregnancy in patients with recurrent miscarriages (173). Another team showed similar results with the analysis of hCG 11 days after embryo transfer (28). hCG-H could also be used as a predictor for progressive pregnancy vs. nonprogressive pregnancy (174).

Recurrent spontaneous abortion (RSA) is one of the most common complications of early pregnancy. This affects about 10–20% of all pregnancies (175). In 80% of cases, spontaneous miscarriages occur during the first 12 weeks of pregnancy. The causes of embryo loss are variable and include cytogenetic abnormalities, maternal problems (e.g., lupus erythematosus or diabetes), uterine malformations, cigarette smoking, or inadequate placental development (176, 177). One study showed that hCG-related cytokines, macrophage inflammatory protein 1 alpha (MIP1a)/hCG, granulocyte-colony stimulating factor (GCSF)/IL-1ra, and MIP1a/TGF-beta1 ratios after 4 weeks of pregnancy were significantly altered in women with spontaneous miscarriages (178). hCG treatment in a mouse model of spontaneous miscarriages increases the number of Treg cells at the maternal/fetal interface and decreases the number of miscarriages. Schumacher et al. suggest that levels of hCG and Treg in the decidual and placenta of pregnant women with RSA are lower than in normal pregnant women (90).

A meta-analysis of five studies was conducted to determine whether hCG treatments could prevent miscarriage in patients. The results of this study show that there is a nonsignificant beneficial trend for women with RSA to receive hCG in early pregnancy. However, this result remains ambiguous and therefore does not demonstrate the interest of treating patients with hCG in the event of a history of miscarriages (176). The combination of hCG and immunoglobulin treatment on Th17+ cells and Foxp3+ Treg cells in patients with RSA was analyzed, and the Th17/Treg ratio was decreased, which could be beneficial for these patients (179). Another study investigated the impact of hCG in the regulation of FOXP3+ Treg cells in patients with RSA and may have a positive impact in these patients (155).

## Conclusions

hCG is involved in many processes ensuring the smooth progress of a pregnancy: the recognition of the pregnancy by the maternal organism, the maintenance of the corpus luteum, the stimulation progesterone production, the strengthening of the implantation of the embryo, angiogenesis, and vasculogenesis necessary for placental development, control of trophoblast differentiation, and finally immune regulation in the maternal/fetal interface.

These different actions have allowed this hormone to be considered as a treatment in cases of RIF and RSA. The future will tell if hCG is an effective tool to help these patients to improve their uterine receptivity. We believe that patient populations need to be more targeted to study hCG to have a beneficial effect from this hormone. Targeting must focus on the immunological and angiogenic profile of the patients to detect their specific problems.

## Author Contributions

VGr has written the article with the help of SP. VGe, BP, MN, and J-MF have corrected the article and made remarks and suggestions.

### Conflict of Interest

The authors declare that the research was conducted in the absence of any commercial or financial relationships that could be construed as a potential conflict of interest.
